# Sustained Improvement in Pain with Talar OsteoPeriostic Grafting from the Iliac Crest (TOPIC) for Medial Osteochondral Lesions of the Talus: A Concise 5-Year Follow-up of a Previous Report*

**DOI:** 10.2106/JBJS.24.01377

**Published:** 2025-12-09

**Authors:** Julian J. Hollander, Jari Dahmen, Sjoerd A.S. Stufkens, Gino M.M.J. Kerkhoffs

**Affiliations:** 1 Department of Orthopedic Surgery and Sports Medicine, Amsterdam UMC, University of Amsterdam, Amsterdam, The Netherlands; 2 Amsterdam Movement Sciences, Programs Sports and Musculoskeletal Health, Amsterdam, The Netherlands; 3 Academic Center for Evidence Based Sports Medicine (ACES), Amsterdam UMC, Amsterdam, The Netherlands; 4 Amsterdam Collaboration for Health and Safety in Sports (ACHSS), International Olympic Committee (IOC) Research Center, Amsterdam UMC, Amsterdam, The Netherlands

## Abstract

Talar OsteoPeriostic grafting from the Iliac Crest (TOPIC) for large osteochondral lesions of the medial talar dome was shown to be effective at 2 years of follow-up. In this prospective cohort study, our aim was to evaluate the clinical and radiographic outcomes at 5 years of follow-up. The primary outcome was the Numeric Rating Scale (NRS) score for pain during walking. Secondary outcomes included NRS scores for pain during rest and during stair-climbing as well as scores on the Foot and Ankle Outcome Score (FAOS) subscales, Short Form (SF)-36 Physical Component Summary and Mental Component Summary scores, and the American Orthopaedic Foot & Ankle Society (AOFAS) ankle-hindfoot score. Computed tomography (CT) scans were evaluated. Thirty-seven patients were included. Two patients died and 2 underwent ankle arthrodesis before 5 years and thus were not included in the 5-year statistical analysis of outcomes. The median NRS score for pain during walking improved from 7 (interquartile range [IQR], 5 to 8) preoperatively to 2 (IQR, 1 to 4) at 5 years (p < 0.001), surpassing the minimal clinically important difference. All secondary clinical outcomes improved significantly. All grafts showed incorporation, and 87% of the patients with a CT scan at 5 years demonstrated evidence of cyst formation. Osteoperiosteal autografting using the TOPIC technique is an effective treatment option for large medial osteochondral lesions of the talus, with durable results at 5 years of follow-up.

**Level of Evidence:**

Therapeutic Level IV. See Instructions for Authors for a complete description of levels of evidence.

For osteochondral lesions of the talus that are large (surface area of >150 mm^2^), including those operatively treated previously (non-primary) or not (primary), osteochondral autograft transplantation therapy might be indicated^1,2^. Previously, most osteochondral autografts were harvested from the ipsilateral knee^3,4^. However, donor-site morbidity was reported in up to 35% of patients undergoing osteochondral harvesting from the knee^3,5-12^. Moreover, the cylindrical grafts may not be sized perfectly to fit the talar defect^13,14^. To prevent donor-site morbidity, allografts may be used (if available). Allografts, however, were shown to fail to incorporate in 11% to 13% of cases, resulting in a high revision rate of 45%^2,15,16^.

The novel technique of Talar OsteoPeriostic grafting from the Iliac Crest (TOPIC) aims to overcome these disadvantages through use of an osteoperiosteal autograft from the ipsilateral iliac crest, including the overlying periosteal layer^17^. The curvature of the iliac crest matches the curvature of the talar surface, and the periosteum might be beneficial, as the cambium layer has chondrogenic potential^18-22^.

We found that TOPIC had good clinical results at 2 years of follow-up, as previously reported^23^. Osteochondral autografts have been shown to have sustained, long-term, moderate to favorable outcomes, but considerable donor-site morbidity^13,24,25^. Regarding osteoperiosteal autografts for osteochondral lesions of the talus, the current literature only focused on follow-up of <4 years or was retrospective in nature^3,26-29^. It is unknown whether clinically relevant improvement is sustained at longer follow-up^23^. Therefore, the primary aim of the present study was to assess the Numeric Rating Scale (NRS) score for pain during walking at 5 years following TOPIC for the treatment of large medial osteochondral lesions of the talus. The secondary aim was to assess other clinical and radiographic outcomes following the TOPIC procedure. We hypothesized that the effectiveness of the TOPIC procedure for such lesions would be sustained, with low donor-site morbidity.

## Materials and Methods

This was a single-center, prospective cohort study performed in a tertiary referral academic center that is recognized nationally and internationally for its expertise in the treatment of osteochondral lesions of the foot and ankle. The study was approved by the Medical Ethics Committee (reference number: W14_237), and all patients provided informed consent. The STROBE (Strengthening the Reporting of Observational Studies in Epidemiology) checklist was used as a guideline for the present study^30^.

### Patient Selection

Patients who underwent the press-fit TOPIC procedure for a large medial osteochondral lesion of the talus, with follow-up of at least 5 years, were eligible for inclusion in the present study. Patients with concomitant injuries or conditions (e.g., rheumatoid arthritis, advanced osteoporosis, bacterial arthritis, or malignancies), advanced osteoarthritis (Kellgren-Lawrence grade III or IV^31^), or a metal implant in their patient history (e.g., HemiCAP; Arthrosurface) were excluded.

### Treatment

All patients were operated on by 2 fellowship-trained foot and ankle surgeons using the previously published surgical technique for medial lesions^17^. The indication for the procedure was a large (i.e., >10-mm anteroposterior or mediolateral diameter or depth) symptomatic osteochondral lesion of the talus. Both primary lesions and non-primary lesions were included. For medial lesions, a closed epiphyseal growth plate is needed in order to allow a medial malleolar osteotomy^17^.

### Outcomes Assessment

All patient characteristics and demographics, such as sex, age at the time of surgery, body mass index (BMI), and smoking status were extracted.

#### Clinical Outcomes

All clinical outcomes were gathered preoperatively and at 1, 2, and 5 years postoperatively by independent researchers. The primary outcome in the present study was the NRS score for pain during walking at 5 years of follow-up^32^. Failure was defined as the need for ankle arthrodesis due to persistent patient-reported pain and/or functional limitations.

Secondary patient-reported outcome measures (PROMs) included NRS scores for pain during rest and during stair-climbing. In addition, the 5 subscales of the validated Foot and Ankle Outcome Score (FAOS) ^33-35^, the Physical Component Summary (PCS) and Mental Component Summary (MCS) of the Short Form (SF)-36^36^, and the American Orthopaedic Foot & Ankle Society (AOFAS) ankle-hindfoot score^37^ were assessed.

#### Radiographic Evaluation

Radiographic evaluation was performed using computed tomography (CT) scans preoperatively and at 3 months and 1, 2, and 5 years postoperatively.

Baseline lesion characteristics, including the surface area (mediolateral diameter [ML] × anteroposterior diameter [AP] × 0.79)^38^, volume (ML × AP × depth)^38^, morphology (classified as fragmentary, cystic, and crater-like)^39^, location (using a 9-zone grid)^40^, and osteoarthritis grade^54^ were evaluated independently by 2 authors. In cases of disagreement, a discussion was held after the primary assessment. If no consensus was reached after a discussion, the senior author’s evaluation was decisive.

#### Complications and Additional Procedures

All complications were assessed as previously defined by Sokol and Wilson, namely: “any undesirable, unintended, and direct result of an operation affecting the patient.”^12,41^ In addition, the hardware removal rate and time to hardware removal and frequency of donor-site morbidity (i.e., pain, disability, or hindrance related to the donor site or the surrounding area affecting activities in daily living) were assessed^23^. Furthermore, the number of additional graft-related procedures was documented.

### Statistical Analysis

Dichotomous and categorical variables are reported as absolute numbers with percentages. Continues variables are reported as the mean and standard deviation (SD), or as the median and interquartile range (IQR) for non-normally distributed data. Normality was assessed using visual inspection of box plots and using the Shapiro-Wilk test. Pre- and postoperative data were compared using the paired t test (normally distributed data) or the Wilcoxon signed-rank test (non-normally distributed data). All statistical analyses were performed using custom-made scripts in Python (version 3.11.4; Python Software Foundation)^42^ with the packages SciPy^43^ and Matplotlib.

To calculate the minimal sample size needed for the primary outcome, we performed an a priori power analysis using G*Power (version 3.1.9.6; University of Düsseldorf)^55^. For the calculation, a power of 90% with α = 0.05 was used. A minimal clinically important difference (MCID) of 2.0 (SD, 2.5) was used, in line with previous studies^44-46^. On the basis of the calculation, at least 20 patients were needed for inclusion.

## Results

### Patient Selection and Characteristics

**Fig. 1:**
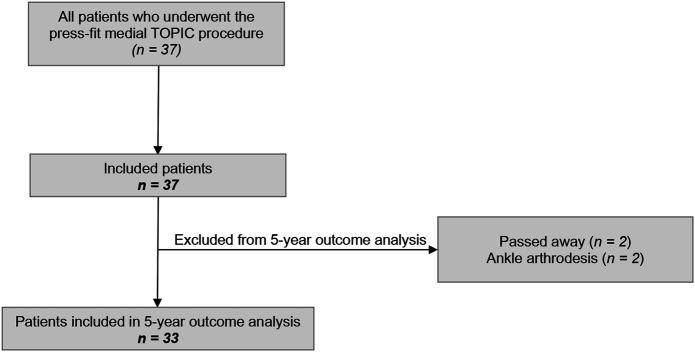
Flowchart of patient selection.

A total of 37 patients were included in the present study (Fig. 1). Two patients died before 5 years of follow-up, both unrelated to the surgery. Another 2 (5%) of the patients were considered to have had failure of TOPIC, as they underwent ankle arthrodesis due to persistent pain and/or functional complaints, at 4 years and at 4 years and 9 months of follow-up, respectively. Thus, while these 4 patients were included in the analysis of 2-year outcomes within the present study, they were not included in the statistical analysis of the 5-year outcomes; a total of 33 patients were analyzed at the 5-year follow-up. Kaplan-Meier analysis demonstrated a survival rate of 94% at 5 years (see Appendix).

Three patients did not visit the outpatient clinic and thus were not available for radiographic follow-up, but they did fill out the questionnaires and are included in the analyses of clinical outcomes, complications, and additional procedures. Patient and lesion characteristics at baseline are summarized in Table 1.

**Table 1:** Patient and Lesion Characteristics at Baseline*

Patient characteristics	
Age at surgery *(yr)*	31 [22-39]
Follow-up *(mo)*	64 [61-70]†
Sex: male/female	12 (32%)/25 (68%)
BMI *(kg/m*^*2*^*)*	26 [24-30]
Laterality: right/left	20 (54%)/17 (46%)
Smoking: yes/no	10 (27%)/27 (73%)
No. (%) of patients with concomitant procedures during TOPIC	2 (5%)‡
Lesion characteristics	
Nature: primary/non-primary	12 (32%)/25 (68%)
Mean no. of previous surgeries per patient	0.9
Lesion size	
Anteroposterior diameter *(mm)*	18 [17-20]
Mediolateral diameter *(mm)*	13 [10-15]
Depth diameter *(mm)*	10 [9-13]
Surface area *(mm*^*2*^*)*	177 [150-243]
Volume *(cm*^*3*^*)*	2.4 [1.8-3.7]
Lesion location	
Zone 4	29 (78%)
Zone 7	8 (22%)
Lesion morphology	
Crater	19 (51%)
Cystic	16 (43%)
Fragmentary	2 (5%)
Preop. ankle osteoarthritis grade	
0	0 (0%)
1	26 (70%)
2	11 (30%)
3	0 (0%)
Preop. osteophytes	
No. of patients	36 (97%)
Total no. of osteophytes	68
Mean no. of osteophytes per patient	1.8

* Continuous variables are given as the median, with the interquartile range in square brackets, except where otherwise indicated; dichotomous and categorical variables are given as the number with the percentage in parentheses.

† Excluding the 4 patients who did not reach 5-year follow-up.

‡ One Duquennoy procedure and 1 supramalleolar osteotomy.

### Clinical Outcomes

#### Primary Outcome

**Fig. 2:**
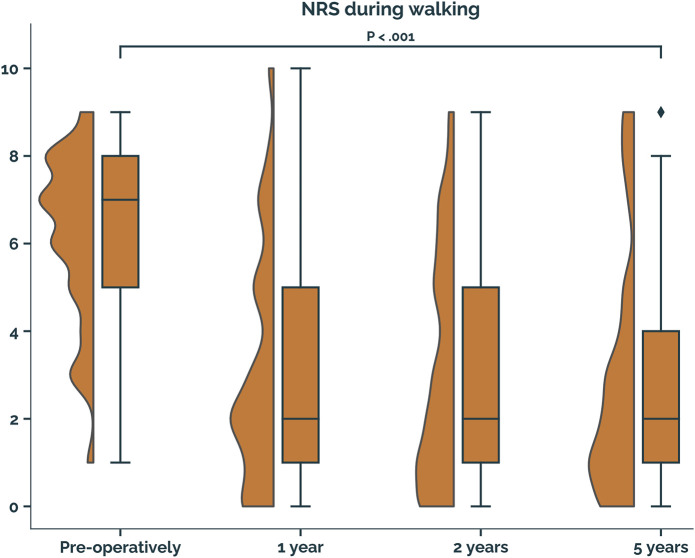
Half-violin and boxplots of Numeric Rating Scale (NRS) scores during walking preoperatively and at 1 year, 2 years, and 5 years of follow-up. The interquartile range (IQR) is represented as a box, and the median is indicated as a horizontal line within the box. The whiskers extend to the highest and lowest values within 1.5-times the IQR. Data points outside of this range are indicated with a diamond marker. The half-violin plot to the left of each boxplot represents the density distribution of the data.

The median NRS score for pain during walking, the primary outcome, improved from 7 (IQR, 5 to 8) preoperatively to 2 (IQR, 1 to 4) at 5 years of follow-up (p < 0.001) (Fig. 2).

#### Secondary Clinical Outcomes

Median NRS scores for pain during rest and during stair-climbing both decreased significantly from preoperatively to 5 years: from 3 (IQR, 2 to 6) to 1 (IQR, 0 to 2) for pain during rest and from 7 (IQR, 4 to 7) to 2 (IQR, 1 to 4) for pain during stair-climbing. Moreover, all FAOS subscale scores improved significantly, as did the AOFAS, and scores for both the SF-36 MCS and PCS. All secondary clinical outcomes are presented in Table 2, with boxplots shown in the Appendix.

**Table 2:** Secondary Clinical Outcomes

Outcome	Median [IQR]		P Value
	Preop.	5-Yr	
NRS			
Pain during rest	3 [2-6]	1 [0-2]	<0.001
Pain during stair-climbing	7 [4-7]	2 [1-4]	<0.001
AOFAS	53 [44-67]	87 [82-100]	<0.001
FAOS			
Symptoms	50 [39-64]	64 [46-79]	0.008
Pain	53 [44-61]	78 [64-92]	<0.001
Activities of daily living	65 [51-74]	93 [75-97]	<0.001
Sports	25 [20-40]	60 [45-80]	<0.001
Quality of life	19 [6-31]	44 [38-56]	<0.001
SF-36			
PCS	32 [30-35]	34 [33-37]	0.02
MCS	41 [35-47]	47 [40-51]	0.001

#### Radiographic Outcomes

All patients (100%) received a preoperative CT scan. Postoperatively, 37 (100%) received a CT scan at 3 months, 36 (97%) at 1 year, 37 (100%) at 2 years, and 30 (91% of the 33 included patients) at 5 years.

All patients (100%) showed graft incorporation and union at the osteotomy site at 3 months. At 2 years, 28 (76% of 37 patients) demonstrated the presence of postoperative cyst development. At 5 years, 26 (87% of the 30 patients with a CT scan) showed cyst presence (Table 3). Three sample cases are presented in the Appendix.

**Table 3:** Radiographic Outcomes

	3 Mo (N = 37)	1 Yr (N = 36)	2 Yr (N = 37)	5 Yr (N = 30)
Union at the osteotomy site	32 (100%)	—	—	—
Graft incorporation	32 (100%)	36 (100%)	37 (100%)	30 (100%)
Cyst presence: yes/no	—	19 (53%)/17 (47%)	28 (76%)/9 (24%)	26 (87%)/4 (13%)

#### Complications and Additional Procedures

Among the 37 patients, 1 complication (3%) occurred: temporary hypoesthesia of the superficial peroneal nerve. No patients reported issues suggesting donor-site morbidity. In 16 (43%) of the patients, medial malleolar screws were removed at a median time of 16.4 months postoperatively. Four (11%) of the patients underwent an additional graft-related procedure, all being arthroscopic shaping of the graft.

## Discussion

The main finding of the present study was that the clinical effectiveness of the TOPIC procedure was sustained with respect to the primary patient outcome, with the improvement in the median NRS score for pain during walking at 5 years (2 [IQR, 1 to 4]), compared with preoperatively (7 [IQR, 5 to 8]), exceeding the MCID. We also observed improvement in all other clinical outcomes. With respect to radiographic outcomes, 100% of the grafts showed incorporation, with 87% of patients having cysts present in or around the graft. No patients reported issues suggesting donor-site morbidity.

The observed improvement in the NRS score for pain during walking is in line with the excellent outcomes seen in osteoperiosteal autografting. A recent retrospective study by Li et al.^29^ investigated osteoperiosteal grafting from the tibia. That study observed a similar improvement in pain scores, as they reported a decrease in long-term (>5 years) scores from 6 to 2 on a visual analog scale. However, 91% of the patients had a primary osteochondral lesion of the talus, compared with only 32% of the patients in the present study having a lesion that was primary in nature. Moreover, in the present study, we used an en-bloc graft instead of cylindrical grafts. In comparison with other, established procedures for large osteochondral lesions of the talus, the postoperative PROM scores observed in this study can be considered similar. In the case of osteochondral allograft use, improvement in the AOFAS from 52 to 84 was reported by Adams et al.^47^ at a mean follow-up of 55 months. Additionally, in a study on the use of autologous chondrocyte implantation, a postoperative AOFAS of 85.9 was reported at a follow-up of 2 to 10 years^48^. However, in a study of ankle arthrodesis, which can be used as the primary treatment for large osteochondral lesions of the talus or as a salvage procedure, AOFAS values of 68.9 (open) and 78.9 (arthroscopic) were reported at 2 years of follow-up^49^. In contrast, in the present study, we report a score of 87 at 5 years, despite postoperative pain scores similar to those following arthrodesis, possibly suggesting superior long-term functional outcomes.

The presence of cysts increased over the years, to a rate of 87% (26 of 30 patients) at 5 years of follow-up, up from 76% (28 of 37) at 2 years. In our study on the 2-year follow-up, no relation between the presence of cysts and pain during walking was found^23^. The rate of patients with postoperative cysts is in line with other studies reporting on this following osteochondral or osteoperiosteal autografting for osteochondral lesions of the talus^3,29,50^. A recent systematic review did not identify a relationship between cyst formation and clinical symptoms^51^. The relationship between cysts and clinical failure has thus not been established, and therefore cyst development is not currently considered failure, but it remains under investigation. Potentially, cysts become symptomatic over the longer term, and thus longer follow-up studies are needed. However, cysts may also merely remain an asymptomatic radiographic finding. Potential contributing factors to cyst development include small-crack formation when impacting the press-fit graft, or failure of subchondral bone to communicate adequately with the joint space, thereby allowing the formation of bone channels^52,53^. The recurrence of cysts in the graft may support a hypothesis that cystic osteochondral lesions of the talus are more likely the result of repetitive overload than a single traumatic event. Further investigations, such as analyses of gait and intra-articular pressure distribution, are needed.

Regarding the ingrowth of the grafts, all grafts (100%) showed incorporation on postoperative CT scans. In comparison, failure rates of 11% to 13% have been described for osteochondral allografts^15,16^. When using autografts, however, donor-site morbidity may be a complication. In the present study, no patient reported issues suggesting donor-site morbidity (of the iliac crest), while the literature shows rates of up to 35% when grafts are harvested from the knee^3,5-12^.

The present study had some limitations. We did not investigate any potential factors influencing the outcomes because of the limited sample size and, thus, low statistical power for such analyses. Moreover, the cases of failure were not included in the 5-year outcome analysis as no questionnaires at the time of failure were available and a last-observation-carried-forward methodology was considered inadequate (as the 2-year outcome was still good for these patients).

Among the study’s strengths are its prospective nature, the a priori power analysis, and 100% clinical and 91% radiographic follow-up among the 33 patients included in the 5-year analysis.

The clinical relevance of the present study is that it is the first study, to our knowledge, to report on 5-year outcomes following osteoperiosteal autografting from the iliac crest for medial osteochondral lesions of the talus. The indication for a TOPIC procedure is a symptomatic primary or non-primary medial osteochondral lesion of the talus that is >10 mm in diameter (anteroposterior or mediolateral) and that has not responded to nonoperative treatment^17^. For these lesions, the present study showed durable effective results. Contraindications are advanced osteoarthritis, multiple osteochondral lesions of the talus, malignancies, or active infectious ankle pathology^17^.

Future research should focus on identifying prognostic factors of postoperative outcomes on the basis of preoperative patient and lesion characteristics and should assess the effectiveness of the TOPIC procedure in other centers. The present study can be used as a guideline to statistically power these studies.

### Conclusions

Osteoperiosteal autografting using the TOPIC procedure is an effective treatment option for large medial osteochondral lesions of the talus, with durable results at 5 years of follow-up, as shown by this prospective series. The median score for pain during walking improved from 7 (IQR, 5 to 8) preoperatively to 2 (IQR, 1 to 4) at the 5-year postoperative follow-up, thereby surpassing the MCID.

## Appendix

Supporting material provided by the authors is posted with the online version of this article as a data supplement at jbjs.org (http://links.lww.com/JBJS/I994).
